# (20S*,24S*)-25-Hy­droxy-20,24-ep­oxy-*A*-homo-4-oxadammaran-3-one (Chrysura) isolated from the leaves of *Walsura chrysogyne*

**DOI:** 10.1107/S1600536811047337

**Published:** 2011-11-12

**Authors:** Ilya Iryani Mahmod, Huey Chong Kwong, Mohamed Ibrahim Mohamed Tahir, Intan Safinar Ismail

**Affiliations:** a Department of Chemistry, Faculty of Science, Universiti Putra Malaysia, 43400 UPM Serdang, Selangor, Malaysia

## Abstract

The title dammarane triterpenoid, C_30_H_50_O_4_, assigned the name chrysura, was isolated from an ethyl acetate extract of *Walsura chrysogyne* leaves (Meliaceae). It has 20S*,24S* relative stereochemistry and an oxepanone ring with two methyl groups at position 4. The two cyclo­hexane rings adopt chair conformations. The cyclo­pentane and tetra­hydro­furan rings have envelope conformations; their mean planes make a dihedral angle of 13.1 (3)°, indicating that the rings are only slightly tilted with respect to each other. There is an intra­molecular C—H⋯O hydrogen bond in the mol­ecule, which forms *S*(6) and *S*(7) ring motifs. In the crystal, mol­ecules are linked *via* O—H⋯O and C—H⋯O hydrogen bonds, forming chains propagating along [001] which stack along the *b*-axis direction.

## Related literature

For related structures, see: Pan *et al.* (2010 ▶). For graph-set analysis, see: Bernstein *et al.* (1995 ▶). For the biological activity of related compounds, see: Burkill (1966 ▶); Hegnauer (1990 ▶); Fujiwara *et al.* (1982 ▶).
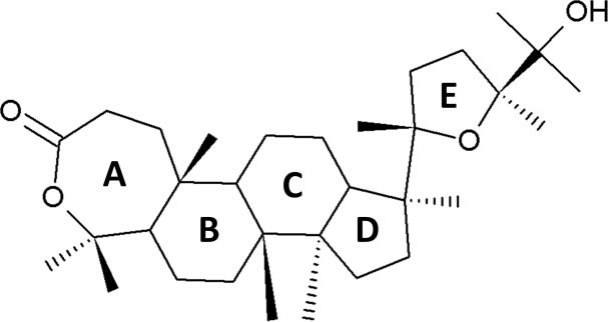


## Experimental

### 

#### Crystal data

C_30_H_50_O_4_
*M* *_r_* = 474.70
Orthorhombic, 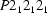

*a* = 6.9881 (1) Å
*b* = 11.0108 (2) Å
*c* = 34.9733 (7) Å
*V* = 2691.01 (8) Å^3^
*Z* = 4
Cu *K*α radiation
μ = 0.59 mm^−1^
*T* = 100 K
0.40 × 0.08 × 0.07 mm

#### Data collection

Bruker APEXII CCD diffractometer
Absorption correction: multi-scan (*SADABS*; Bruker, 2009 ▶) *T*_min_ = 0.801, *T*_max_ = 0.960
48305 measured reflections
5058 independent reflections
5040 reflections with *I* > 2σ(*I*)
*R*_int_ = 0.045

#### Refinement

*R*[*F* ^2^ > 2σ(*F* ^2^)] = 0.092
*wR*(*F* ^2^) = 0.233
*S* = 1.21
5058 reflections
316 parameters
H-atom parameters constrained
Δρ_max_ = 0.47 e Å^−3^
Δρ_min_ = −0.38 e Å^−3^

### 

Data collection: *APEX2* (Bruker, 2009 ▶); cell refinement: *SAINT* (Bruker, 2009 ▶); data reduction: *SAINT*; program(s) used to solve structure: *SHELXS97* (Sheldrick, 2008 ▶); program(s) used to refine structure: *SHELXL97* (Sheldrick, 2008 ▶); molecular graphics: *Mercury* (Macrae *et al.*, 2006 ▶); software used to prepare material for publication: *SHELXL97*.

## Supplementary Material

Crystal structure: contains datablock(s) global, I. DOI: 10.1107/S1600536811047337/su2332sup1.cif

Structure factors: contains datablock(s) I. DOI: 10.1107/S1600536811047337/su2332Isup2.hkl

Additional supplementary materials:  crystallographic information; 3D view; checkCIF report

## Figures and Tables

**Table 1:** Hydrogen-bond geometry (Å, °)

*D*—H⋯*A*	*D*—H	H⋯*A*	*D*⋯*A*	*D*—H⋯*A*
C19—H19*A*⋯O32	0.96	2.44	3.082 (6)	124
O34—H34*A*⋯O32^i^	0.82	2.20	3.010 (5)	170
C26—H26*A*⋯O31^i^	0.96	2.53	3.392 (7)	150

Symmetry code: (i) 
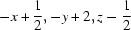
.

**Fig. 1.**
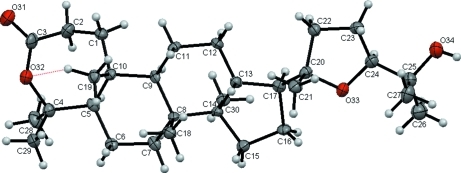
The molecular structure of the title molecule, chrysura (1), showing 50% probability displacement ellipsoids and the atom-numbering scheme. The intramolecular C-H···O hydrogen bond is shown as a dashed red line.

**Fig. 2.**
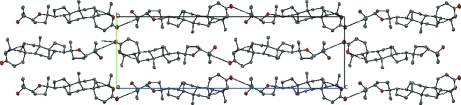
The crystal packing of the title compound, chrysura (1), viewed along the a axis, showing the formation of the hydrogen bonded chains (see Table 1 for details). H atoms not involved in the hydrogen bonds (dashed lines) have been omitted for clarity.

## supplementary crystallographic information

### Comment

Meliaceae or Mahogany is a plant family, in the order of Sapindales, which consists of flowering plants of mostly trees, shrubs and a few herbaceous plants (Burkill, 1966). This family is noted for the wide range of compounds of different classes of which it is compossed, for example, terpenoids (triterpenoids, monoterpenes, sesquiterpenes, limonoids), saponins, alkaloids, polyphenols, quinines, fatty and hydroxyl acids (Hegnauer, 1990). Among these groups of constituents, some are responsible for biological activities such as antiviral, anthelmintic, antitumor, anti-inflammatory and anti-rheumatic, which have been scientifically proven (Fujiwara *et al.*, 1982). *Walsura chrysogyne* is a Meliaceae species which is among the least explored of higher plants.

The title dammarane triterpenoid, namely chrysura (1), has been isolated for the first time from the ethyl acetate extract of the leaves of *Walsura chrysogyne* (Meliaceae). Recently, the same compound was reported to have been obtained from *Aglaia foveolata*, but in resin form (compound 5 in reference Pan *et al.*, 2010). They determined its relative stereochemistry by Nuclear Magnetic Resonance (NMR) spectroscopy. Herein, we describe the crystal structure of the title compound, chrysura (1), whose relative configuration was also obtained by two-dimensional NMR spectroscopy. By a close comparison of the ^13^C NMR signals at C-20, C-21, C-22, C-23 and C-24 reported for compound 5 (*δ* 86.5, 27.2, 34.8, 26.3 and 86.4; Pan *et al.*, 2010) and those obtained for the title compound, chrysura (1) (*δ* 86.5, 27.2, 35.0, 26.4 and 86.5), it was shown that these two compounds are identical. This is substantiated by the ^1^H NMR signal at H-24 of chrysura (1), which is a doublet of doublet with J values of 10 and 5.5 Hz, comparable to the values observed for compound 5, that is 9.9 and 5.6 Hz. Hence, the relative configuration at C20 and C24 of chrysura (1), was determined by NMR to be the same as that of compound 5 [Pan *et al.*, 2010].

The molecular structure of the title molecule, chrysura (1), is shown in Fig. 1. The two cyclohexane rings, B (C5-C10) and C (C8,C9,C11-C14), adopt chair conformations. The cyclopentane ring D (C13-C17) and the tetrahydrofuran ring E (O33,C20, C22-C24) have envelope conformations, with atoms C14 and C23 at the flap of rings D and E, respectively. The mean planes through rings D and E make a dihedral angle of 13.1 (3)°, indicating that they are only slightly twisted with respect to each other. As shown in Fig. 1, the structure of the molecule is stabilized by an intramolecular C—H···O hydrogen bond (Table 1), which forms *S*(6) and *S*(7) ring motifs (Bernstein *et al.*, 1995).

In the crystal of chrysura (1), molecules are linked *via* intermolecular O—H···O and C—H···O hydrogen bonds (Table 1), forming chains propagating along [001]. These chains stack along the *b*-axis, as shown in Fig. 2.

Hence, in the title compound, chrysura (1), the relative configurations at C20 and C24 of the epoxy unit (ring E) have been confirmed to be *S*-methyl configurations.

### Experimental

The air-dried ground leaves of *Walsura chrysogyne* (8.94 kg) collected at Pasir Raja, Terengganu, Malaysia, were macerated in methanol at room temperature (3 × 1000 ml). The crude extract (230 g) was partitioned into hexane (12.2 g), ethyl acetate (EtOAc; 16.6 g), and water (16.8 g). A portion (9.0 g) of the EtOAc extract was further fractionated by using vacuum column chromatography on silica gel normal phase (7.5 × 20 cm) eluted with CHCl_3_, and CHCl_3_—MeOH in 10% increasing amounts of MeOH. Fraction MeOH-CHCl_3_ [9:1] (2.0 g) was subjected to another column chromatography on Sephadex LH-20 (2 × 30 cm) with CHCl_3_–MeOH (9:1) to yield four fractions. The fraction obtained by hexane-EtOAc [7:3] (85.3 mg) was further purified on silica gel normal phase (1 × 20 cm) eluted with hexane-acetone (9:1) to afford the title compound (134.8 mg, 0.059%). Colourless needle-shaped crystals of the title compound, suitable for *X-ray* diffraction analysis, were recrystallized from ethyl aceate-acetone. The ^1^H- and ^13^C-NMR spectral data were consistent with those reported by (Pan *et al.*, 2010).

### Refinement

All the H atoms were positioned geometrically and refined using a riding model: O—H = 0.82 Å and C—H = 0.93 – 0.98 Å with *U*_iso_~(H) = 1.5*U*_eq_(O, C_methyl_), and = 1.2*U*_eq_(C) for all other C-bound H atoms. A rotating-group model was applied for the methyl groups. The anomalous dispersion effects of the atoms in the molecule are not sufficient to determine the absolute structure of the molecule in the crystal [Flack parameter = 0.1 (5)].

### Crystal data

**Table:**

C_30_H_50_O_4_	*F*(000) = 1048
*M**_r_* = 474.70	*D*_x_ = 1.172 Mg m^−^^3^
Orthorhombic, *P*2_1_2_1_2_1_	Cu *K*α radiation, λ = 1.54178 Å
Hall symbol: P 2ac 2ab	Cell parameters from 9811 reflections
*a* = 6.9881 (1) Å	θ = 3–69°
*b* = 11.0108 (2) Å	µ = 0.59 mm^−^^1^
*c* = 34.9733 (7) Å	*T* = 100 K
*V* = 2691.01 (8) Å^3^	Needle, colourless
*Z* = 4	0.40 × 0.08 × 0.07 mm

### Data collection

**Table:**

Bruker APEXII CCD diffractometer	5058 independent reflections
Radiation source: fine-focus sealed tube	5040 reflections with *I* > 2σ(*I*)
graphite	*R*_int_ = 0.045
φ and ω scans	θ_max_ = 69.9°, θ_min_ = 2.5°
Absorption correction: multi-scan (*SADABS*; Bruker, 2009)	*h* = −7→8
*T*_min_ = 0.801, *T*_max_ = 0.960	*k* = −13→13
48305 measured reflections	*l* = −41→42

### Refinement

**Table:**

Refinement on *F*^2^	Hydrogen site location: inferred from neighbouring sites
Least-squares matrix: full	H-atom parameters constrained
*R*[*F*^2^ > 2σ(*F*^2^)] = 0.092	*w* = 1/[σ^2^(*F*_o_^2^) + (0.0677*P*)^2^ + 8.7996*P*] where *P* = (*F*_o_^2^ + 2*F*_c_^2^)/3
*wR*(*F*^2^) = 0.233	(Δ/σ)_max_ < 0.001
*S* = 1.21	Δρ_max_ = 0.47 e Å^−^^3^
5058 reflections	Δρ_min_ = −0.38 e Å^−^^3^
316 parameters	Extinction correction: *SHELXL97* (Sheldrick, 2008), Fc^*^=kFc[1+0.001xFc^2^λ^3^/sin(2θ)]^-1/4^
0 restraints	Extinction coefficient: 0.0021 (7)
Primary atom site location: structure-invariant direct methods	Absolute structure: Flack, H. D. (1983). *Acta Cryst.* A39, 876–881
Secondary atom site location: difference Fourier map	Flack parameter: 0.1 (5)

### Special details

**Table:**

Experimental. The needle-shape crystal was placed in the cold stream of an Oxford Cyrosystems Cobra open-flow nitrogen cryostat (Cosier & Glazer, 1986) operating at 100.0 (1) K.
Geometry. All esds (except the esd in the dihedral angle between two l.s. planes) are estimated using the full covariance matrix. The cell esds are taken into account individually in the estimation of esds in distances, angles and torsion angles; correlations between esds in cell parameters are only used when they are defined by crystal symmetry. An approximate (isotropic) treatment of cell esds is used for estimating esds involving l.s. planes.

### Fractional atomic coordinates and isotropic or equivalent isotropic displacement parameters (Å^2^)

**Table:**

	*x*	*y*	*z*	*U*_iso_*/*U*_eq_	
O31	0.0510 (6)	0.8536 (4)	1.00267 (11)	0.0404 (10)	
O32	0.2497 (5)	0.9976 (3)	0.98533 (9)	0.0275 (8)	
O33	0.3411 (5)	1.0371 (3)	0.66313 (9)	0.0277 (8)	
O34	0.2551 (6)	0.9264 (3)	0.56801 (10)	0.0345 (9)	
H34A	0.2593	0.9556	0.5464	0.052*	
C1	0.1697 (7)	0.8667 (4)	0.90988 (13)	0.0237 (10)	
H1A	0.0311	0.8708	0.9109	0.028*	
H1B	0.2030	0.8116	0.8893	0.028*	
C2	0.2412 (9)	0.8102 (5)	0.94789 (14)	0.0306 (12)	
H2A	0.1914	0.7284	0.9505	0.037*	
H2B	0.3799	0.8056	0.9477	0.037*	
C3	0.1774 (8)	0.8847 (5)	0.98059 (13)	0.0287 (12)	
C4	0.4229 (7)	1.0448 (5)	0.96570 (13)	0.0236 (10)	
C5	0.4357 (7)	1.0211 (4)	0.92154 (13)	0.0209 (10)	
H5A	0.5138	0.9478	0.9185	0.025*	
C6	0.5520 (6)	1.1256 (4)	0.90319 (13)	0.0203 (10)	
H6A	0.4782	1.2001	0.9046	0.024*	
H6B	0.6691	1.1376	0.9176	0.024*	
C7	0.6018 (7)	1.0995 (4)	0.86170 (13)	0.0204 (9)	
H7A	0.6752	1.0249	0.8603	0.024*	
H7B	0.6808	1.1648	0.8518	0.024*	
C8	0.4216 (6)	1.0873 (4)	0.83692 (12)	0.0183 (9)	
C9	0.2945 (6)	0.9860 (4)	0.85516 (12)	0.0165 (9)	
H9A	0.3707	0.9117	0.8529	0.020*	
C10	0.2470 (7)	0.9967 (4)	0.89916 (12)	0.0195 (9)	
C11	0.1148 (6)	0.9619 (4)	0.83075 (13)	0.0190 (9)	
H11A	0.0369	1.0348	0.8302	0.023*	
H11B	0.0400	0.8981	0.8426	0.023*	
C12	0.1632 (7)	0.9243 (4)	0.78936 (13)	0.0208 (10)	
H12A	0.2262	0.8458	0.7894	0.025*	
H12B	0.0463	0.9173	0.7746	0.025*	
C13	0.2941 (7)	1.0189 (4)	0.77122 (12)	0.0203 (10)	
H13A	0.2229	1.0956	0.7717	0.024*	
C14	0.4758 (6)	1.0405 (4)	0.79509 (13)	0.0167 (9)	
C15	0.5831 (7)	1.1312 (4)	0.76941 (13)	0.0235 (10)	
H15A	0.7189	1.1311	0.7751	0.028*	
H15B	0.5337	1.2128	0.7728	0.028*	
C16	0.5460 (7)	1.0854 (5)	0.72793 (13)	0.0217 (10)	
H16A	0.6548	1.0393	0.7187	0.026*	
H16B	0.5246	1.1535	0.7108	0.026*	
C17	0.3625 (6)	1.0024 (4)	0.72988 (13)	0.0191 (10)	
H17A	0.4059	0.9182	0.7274	0.023*	
C30	0.5988 (7)	0.9245 (4)	0.79638 (13)	0.0220 (10)	
H30A	0.6375	0.9032	0.7709	0.033*	
H30B	0.5254	0.8593	0.8072	0.033*	
H30C	0.7101	0.9388	0.8118	0.033*	
C19	0.0918 (7)	1.0887 (4)	0.90822 (13)	0.0226 (10)	
H19A	0.0491	1.0775	0.9341	0.034*	
H19B	−0.0139	1.0775	0.8910	0.034*	
H19C	0.1421	1.1693	0.9053	0.034*	
C20	0.2217 (7)	1.0257 (4)	0.69762 (13)	0.0202 (10)	
C21	0.1120 (7)	1.1431 (5)	0.70141 (14)	0.0261 (11)	
H21A	0.2003	1.2093	0.7042	0.039*	
H21B	0.0308	1.1393	0.7235	0.039*	
H21C	0.0353	1.1554	0.6790	0.039*	
C22	0.0884 (7)	0.9175 (5)	0.68812 (14)	0.0245 (10)	
H22A	0.1426	0.8414	0.6970	0.029*	
H22B	−0.0370	0.9283	0.6995	0.029*	
C23	0.0774 (8)	0.9211 (4)	0.64427 (14)	0.0255 (10)	
H23A	0.0413	0.8428	0.6338	0.031*	
H23B	−0.0120	0.9824	0.6355	0.031*	
C24	0.2804 (7)	0.9535 (4)	0.63416 (14)	0.0257 (11)	
H24A	0.3588	0.8800	0.6362	0.031*	
C25	0.3219 (8)	1.0139 (5)	0.59506 (15)	0.0294 (11)	
C26	0.5357 (9)	1.0307 (6)	0.59086 (17)	0.0382 (14)	
H26A	0.5632	1.0659	0.5664	0.057*	
H26B	0.5981	0.9533	0.5929	0.057*	
H26C	0.5815	1.0835	0.6107	0.057*	
C27	0.2159 (9)	1.1313 (5)	0.58997 (15)	0.0351 (13)	
H27A	0.2411	1.1632	0.5649	0.053*	
H27B	0.2579	1.1886	0.6089	0.053*	
H27C	0.0811	1.1173	0.5928	0.053*	
C28	0.5949 (8)	0.9895 (6)	0.98605 (15)	0.0344 (12)	
H28A	0.5901	1.0098	1.0127	0.052*	
H28C	0.7106	1.0210	0.9751	0.052*	
H28D	0.5923	0.9028	0.9832	0.052*	
C29	0.4088 (9)	1.1798 (5)	0.97708 (14)	0.0302 (12)	
H29C	0.3941	1.1862	1.0043	0.045*	
H29D	0.3003	1.2159	0.9647	0.045*	
H29A	0.5232	1.2213	0.9694	0.045*	
C18	0.3211 (7)	1.2099 (4)	0.83435 (14)	0.0237 (10)	
H18A	0.2779	1.2337	0.8593	0.036*	
H18B	0.2133	1.2036	0.8174	0.036*	
H18C	0.4087	1.2696	0.8247	0.036*	

### Atomic displacement parameters (Å^2^)

**Table:**

	*U*^11^	*U*^22^	*U*^33^	*U*^12^	*U*^13^	*U*^23^
O31	0.043 (2)	0.049 (2)	0.029 (2)	−0.013 (2)	0.0095 (18)	0.0026 (18)
O32	0.0313 (18)	0.0309 (19)	0.0202 (15)	−0.0033 (17)	0.0022 (14)	0.0007 (14)
O33	0.0307 (18)	0.0328 (19)	0.0195 (16)	−0.0063 (15)	0.0047 (14)	0.0008 (15)
O34	0.046 (2)	0.035 (2)	0.0225 (17)	−0.0042 (19)	−0.0020 (17)	0.0001 (15)
C1	0.021 (2)	0.028 (3)	0.022 (2)	−0.006 (2)	−0.0023 (19)	0.001 (2)
C2	0.036 (3)	0.031 (3)	0.025 (2)	−0.006 (2)	0.002 (2)	0.006 (2)
C3	0.034 (3)	0.036 (3)	0.017 (2)	−0.002 (2)	−0.005 (2)	0.010 (2)
C4	0.025 (2)	0.027 (2)	0.020 (2)	−0.001 (2)	−0.003 (2)	0.0022 (19)
C5	0.019 (2)	0.022 (2)	0.021 (2)	0.0041 (19)	−0.0062 (19)	−0.0019 (19)
C6	0.012 (2)	0.028 (2)	0.021 (2)	0.0015 (19)	−0.0016 (17)	−0.0021 (19)
C7	0.016 (2)	0.023 (2)	0.022 (2)	0.0068 (19)	−0.0033 (18)	−0.0054 (18)
C8	0.016 (2)	0.022 (2)	0.017 (2)	−0.0077 (19)	0.0012 (18)	−0.0014 (18)
C9	0.011 (2)	0.023 (2)	0.0159 (19)	0.0030 (18)	0.0004 (16)	−0.0053 (18)
C10	0.016 (2)	0.023 (2)	0.019 (2)	−0.0015 (19)	0.0020 (17)	−0.0020 (18)
C11	0.013 (2)	0.021 (2)	0.022 (2)	−0.0026 (17)	−0.0007 (17)	0.0058 (18)
C12	0.021 (2)	0.023 (2)	0.018 (2)	−0.0068 (19)	−0.0034 (18)	−0.0029 (18)
C13	0.024 (2)	0.018 (2)	0.019 (2)	−0.0029 (19)	−0.0040 (19)	0.0013 (17)
C14	0.016 (2)	0.017 (2)	0.017 (2)	−0.0048 (17)	−0.0023 (17)	−0.0003 (17)
C15	0.022 (2)	0.026 (2)	0.023 (2)	−0.004 (2)	0.002 (2)	0.0021 (19)
C16	0.013 (2)	0.028 (2)	0.024 (2)	−0.0043 (19)	0.0007 (18)	0.000 (2)
C17	0.019 (2)	0.015 (2)	0.023 (2)	0.0042 (18)	−0.0006 (18)	−0.0004 (18)
C30	0.016 (2)	0.026 (2)	0.024 (2)	0.0000 (19)	−0.0011 (19)	−0.0006 (19)
C19	0.019 (2)	0.029 (2)	0.020 (2)	0.000 (2)	0.0014 (18)	−0.0009 (19)
C20	0.019 (2)	0.021 (2)	0.021 (2)	0.0000 (19)	−0.0021 (18)	0.0050 (18)
C21	0.022 (2)	0.031 (3)	0.025 (2)	−0.003 (2)	−0.005 (2)	0.004 (2)
C22	0.023 (2)	0.027 (2)	0.023 (2)	−0.001 (2)	0.000 (2)	−0.0035 (19)
C23	0.028 (3)	0.023 (2)	0.026 (2)	−0.007 (2)	0.000 (2)	0.002 (2)
C24	0.030 (3)	0.024 (2)	0.023 (2)	−0.004 (2)	0.000 (2)	0.0011 (19)
C25	0.032 (3)	0.031 (3)	0.025 (2)	−0.002 (2)	0.000 (2)	0.000 (2)
C26	0.040 (3)	0.043 (3)	0.032 (3)	−0.006 (3)	−0.007 (2)	0.007 (3)
C27	0.040 (3)	0.040 (3)	0.025 (2)	0.003 (3)	0.002 (2)	0.007 (2)
C28	0.033 (3)	0.045 (3)	0.025 (2)	0.000 (3)	−0.013 (2)	0.000 (2)
C29	0.034 (3)	0.035 (3)	0.021 (2)	−0.010 (2)	0.002 (2)	−0.006 (2)
C18	0.023 (2)	0.023 (2)	0.025 (2)	−0.002 (2)	0.000 (2)	−0.004 (2)

### Geometric parameters (Å, °)

**Table:**

O31—C3	1.223 (7)	C15—C16	1.558 (6)
O32—C3	1.352 (7)	C15—H15A	0.9700
O32—C4	1.485 (6)	C15—H15B	0.9700
O33—C24	1.433 (6)	C16—C17	1.576 (6)
O33—C20	1.472 (5)	C16—H16A	0.9700
O34—C25	1.428 (6)	C16—H16B	0.9700
O34—H34A	0.8200	C17—C20	1.519 (6)
C1—C2	1.551 (7)	C17—H17A	0.9800
C1—C10	1.576 (7)	C30—H30A	0.9600
C1—H1A	0.9700	C30—H30B	0.9600
C1—H1B	0.9700	C30—H30C	0.9600
C2—C3	1.477 (8)	C19—H19A	0.9600
C2—H2A	0.9700	C19—H19B	0.9600
C2—H2B	0.9700	C19—H19C	0.9600
C4—C28	1.523 (7)	C20—C21	1.509 (7)
C4—C29	1.542 (7)	C20—C22	1.548 (7)
C4—C5	1.569 (6)	C21—H21A	0.9600
C5—C6	1.548 (7)	C21—H21B	0.9600
C5—C10	1.557 (6)	C21—H21C	0.9600
C5—H5A	0.9800	C22—C23	1.536 (6)
C6—C7	1.520 (6)	C22—H22A	0.9700
C6—H6A	0.9700	C22—H22B	0.9700
C6—H6B	0.9700	C23—C24	1.505 (7)
C7—C8	1.535 (6)	C23—H23A	0.9700
C7—H7A	0.9700	C23—H23B	0.9700
C7—H7B	0.9700	C24—C25	1.548 (7)
C8—C18	1.524 (6)	C24—H24A	0.9800
C8—C9	1.562 (6)	C25—C27	1.500 (8)
C8—C14	1.597 (6)	C25—C26	1.513 (8)
C9—C11	1.541 (6)	C26—H26A	0.9600
C9—C10	1.579 (6)	C26—H26B	0.9600
C9—H9A	0.9800	C26—H26C	0.9600
C10—C19	1.518 (6)	C27—H27A	0.9600
C11—C12	1.543 (6)	C27—H27B	0.9600
C11—H11A	0.9700	C27—H27C	0.9600
C11—H11B	0.9700	C28—H28A	0.9600
C12—C13	1.525 (6)	C28—H28C	0.9600
C12—H12A	0.9700	C28—H28D	0.9600
C12—H12B	0.9700	C29—H29C	0.9600
C13—C17	1.533 (6)	C29—H29D	0.9600
C13—C14	1.538 (6)	C29—H29A	0.9600
C13—H13A	0.9800	C18—H18A	0.9600
C14—C15	1.538 (6)	C18—H18B	0.9600
C14—C30	1.540 (6)	C18—H18C	0.9600
			
C3—O32—C4	124.7 (4)	C15—C16—C17	106.4 (4)
C24—O33—C20	110.9 (4)	C15—C16—H16A	110.4
C25—O34—H34A	109.5	C17—C16—H16A	110.4
C2—C1—C10	117.2 (4)	C15—C16—H16B	110.4
C2—C1—H1A	108.0	C17—C16—H16B	110.4
C10—C1—H1A	108.0	H16A—C16—H16B	108.6
C2—C1—H1B	108.0	C20—C17—C13	118.6 (4)
C10—C1—H1B	108.0	C20—C17—C16	113.4 (4)
H1A—C1—H1B	107.2	C13—C17—C16	103.0 (4)
C3—C2—C1	110.1 (4)	C20—C17—H17A	107.0
C3—C2—H2A	109.6	C13—C17—H17A	107.0
C1—C2—H2A	109.6	C16—C17—H17A	107.0
C3—C2—H2B	109.6	C14—C30—H30A	109.5
C1—C2—H2B	109.6	C14—C30—H30B	109.5
H2A—C2—H2B	108.2	H30A—C30—H30B	109.5
O31—C3—O32	116.8 (5)	C14—C30—H30C	109.5
O31—C3—C2	123.5 (5)	H30A—C30—H30C	109.5
O32—C3—C2	119.6 (4)	H30B—C30—H30C	109.5
O32—C4—C28	106.7 (4)	C10—C19—H19A	109.5
O32—C4—C29	99.5 (4)	C10—C19—H19B	109.5
C28—C4—C29	108.4 (4)	H19A—C19—H19B	109.5
O32—C4—C5	116.3 (4)	C10—C19—H19C	109.5
C28—C4—C5	110.4 (4)	H19A—C19—H19C	109.5
C29—C4—C5	114.7 (4)	H19B—C19—H19C	109.5
C6—C5—C10	111.4 (4)	O33—C20—C21	106.7 (4)
C6—C5—C4	108.3 (4)	O33—C20—C17	104.8 (4)
C10—C5—C4	118.4 (4)	C21—C20—C17	114.1 (4)
C6—C5—H5A	106.0	O33—C20—C22	103.3 (4)
C10—C5—H5A	106.0	C21—C20—C22	111.9 (4)
C4—C5—H5A	106.0	C17—C20—C22	114.8 (4)
C7—C6—C5	112.1 (4)	C20—C21—H21A	109.5
C7—C6—H6A	109.2	C20—C21—H21B	109.5
C5—C6—H6A	109.2	H21A—C21—H21B	109.5
C7—C6—H6B	109.2	C20—C21—H21C	109.5
C5—C6—H6B	109.2	H21A—C21—H21C	109.5
H6A—C6—H6B	107.9	H21B—C21—H21C	109.5
C6—C7—C8	111.6 (4)	C23—C22—C20	103.0 (4)
C6—C7—H7A	109.3	C23—C22—H22A	111.2
C8—C7—H7A	109.3	C20—C22—H22A	111.2
C6—C7—H7B	109.3	C23—C22—H22B	111.2
C8—C7—H7B	109.3	C20—C22—H22B	111.2
H7A—C7—H7B	108.0	H22A—C22—H22B	109.1
C18—C8—C7	109.5 (4)	C24—C23—C22	101.1 (4)
C18—C8—C9	113.2 (4)	C24—C23—H23A	111.5
C7—C8—C9	107.4 (4)	C22—C23—H23A	111.5
C18—C8—C14	109.9 (4)	C24—C23—H23B	111.5
C7—C8—C14	110.5 (4)	C22—C23—H23B	111.5
C9—C8—C14	106.2 (3)	H23A—C23—H23B	109.4
C11—C9—C8	111.1 (4)	O33—C24—C23	105.4 (4)
C11—C9—C10	112.4 (3)	O33—C24—C25	107.1 (4)
C8—C9—C10	117.7 (4)	C23—C24—C25	119.1 (4)
C11—C9—H9A	104.7	O33—C24—H24A	108.3
C8—C9—H9A	104.7	C23—C24—H24A	108.3
C10—C9—H9A	104.7	C25—C24—H24A	108.3
C19—C10—C5	112.6 (4)	O34—C25—C27	110.0 (4)
C19—C10—C1	108.2 (4)	O34—C25—C26	109.9 (5)
C5—C10—C1	109.1 (4)	C27—C25—C26	111.7 (5)
C19—C10—C9	113.8 (4)	O34—C25—C24	103.6 (4)
C5—C10—C9	109.0 (4)	C27—C25—C24	112.5 (4)
C1—C10—C9	103.7 (4)	C26—C25—C24	108.8 (4)
C9—C11—C12	112.8 (4)	C25—C26—H26A	109.5
C9—C11—H11A	109.0	C25—C26—H26B	109.5
C12—C11—H11A	109.0	H26A—C26—H26B	109.5
C9—C11—H11B	109.0	C25—C26—H26C	109.5
C12—C11—H11B	109.0	H26A—C26—H26C	109.5
H11A—C11—H11B	107.8	H26B—C26—H26C	109.5
C13—C12—C11	109.8 (4)	C25—C27—H27A	109.5
C13—C12—H12A	109.7	C25—C27—H27B	109.5
C11—C12—H12A	109.7	H27A—C27—H27B	109.5
C13—C12—H12B	109.7	C25—C27—H27C	109.5
C11—C12—H12B	109.7	H27A—C27—H27C	109.5
H12A—C12—H12B	108.2	H27B—C27—H27C	109.5
C12—C13—C17	119.9 (4)	C4—C28—H28A	109.5
C12—C13—C14	112.1 (4)	C4—C28—H28C	109.5
C17—C13—C14	105.8 (4)	H28A—C28—H28C	109.5
C12—C13—H13A	106.0	C4—C28—H28D	109.5
C17—C13—H13A	106.0	H28A—C28—H28D	109.5
C14—C13—H13A	106.0	H28C—C28—H28D	109.5
C13—C14—C15	100.7 (4)	C4—C29—H29C	109.5
C13—C14—C30	110.4 (4)	C4—C29—H29D	109.5
C15—C14—C30	106.5 (4)	H29C—C29—H29D	109.5
C13—C14—C8	110.6 (4)	C4—C29—H29A	109.5
C15—C14—C8	116.2 (4)	H29C—C29—H29A	109.5
C30—C14—C8	111.9 (4)	H29D—C29—H29A	109.5
C14—C15—C16	104.6 (4)	C8—C18—H18A	109.5
C14—C15—H15A	110.8	C8—C18—H18B	109.5
C16—C15—H15A	110.8	H18A—C18—H18B	109.5
C14—C15—H15B	110.8	C8—C18—H18C	109.5
C16—C15—H15B	110.8	H18A—C18—H18C	109.5
H15A—C15—H15B	108.9	H18B—C18—H18C	109.5
			
C10—C1—C2—C3	63.3 (6)	C17—C13—C14—C15	44.3 (4)
C4—O32—C3—O31	169.9 (4)	C12—C13—C14—C30	64.5 (5)
C4—O32—C3—C2	−15.1 (7)	C17—C13—C14—C30	−67.9 (5)
C1—C2—C3—O31	107.2 (6)	C12—C13—C14—C8	−59.9 (5)
C1—C2—C3—O32	−67.5 (6)	C17—C13—C14—C8	167.7 (4)
C3—O32—C4—C28	−76.9 (5)	C18—C8—C14—C13	−63.3 (5)
C3—O32—C4—C29	170.5 (4)	C7—C8—C14—C13	175.7 (4)
C3—O32—C4—C5	46.8 (6)	C9—C8—C14—C13	59.5 (5)
O32—C4—C5—C6	150.4 (4)	C18—C8—C14—C15	50.6 (5)
C28—C4—C5—C6	−87.9 (5)	C7—C8—C14—C15	−70.4 (5)
C29—C4—C5—C6	34.9 (6)	C9—C8—C14—C15	173.5 (4)
O32—C4—C5—C10	22.4 (6)	C18—C8—C14—C30	173.2 (4)
C28—C4—C5—C10	144.1 (4)	C7—C8—C14—C30	52.2 (5)
C29—C4—C5—C10	−93.1 (5)	C9—C8—C14—C30	−64.0 (4)
C10—C5—C6—C7	−58.1 (5)	C13—C14—C15—C16	−38.9 (4)
C4—C5—C6—C7	170.0 (4)	C30—C14—C15—C16	76.3 (4)
C5—C6—C7—C8	62.3 (5)	C8—C14—C15—C16	−158.3 (4)
C6—C7—C8—C18	67.2 (5)	C14—C15—C16—C17	20.4 (5)
C6—C7—C8—C9	−56.1 (5)	C12—C13—C17—C20	74.5 (6)
C6—C7—C8—C14	−171.5 (4)	C14—C13—C17—C20	−157.7 (4)
C18—C8—C9—C11	62.4 (5)	C12—C13—C17—C16	−159.3 (4)
C7—C8—C9—C11	−176.6 (3)	C14—C13—C17—C16	−31.5 (4)
C14—C8—C9—C11	−58.3 (4)	C15—C16—C17—C20	136.0 (4)
C18—C8—C9—C10	−69.2 (5)	C15—C16—C17—C13	6.5 (5)
C7—C8—C9—C10	51.7 (5)	C24—O33—C20—C21	−113.7 (4)
C14—C8—C9—C10	170.0 (4)	C24—O33—C20—C17	125.0 (4)
C6—C5—C10—C19	−78.2 (5)	C24—O33—C20—C22	4.4 (5)
C4—C5—C10—C19	48.3 (6)	C13—C17—C20—O33	164.2 (4)
C6—C5—C10—C1	161.6 (4)	C16—C17—C20—O33	43.1 (5)
C4—C5—C10—C1	−71.9 (5)	C13—C17—C20—C21	47.9 (6)
C6—C5—C10—C9	49.0 (5)	C16—C17—C20—C21	−73.2 (5)
C4—C5—C10—C9	175.6 (4)	C13—C17—C20—C22	−83.2 (5)
C2—C1—C10—C19	−102.1 (5)	C16—C17—C20—C22	155.8 (4)
C2—C1—C10—C5	20.8 (6)	O33—C20—C22—C23	−27.2 (5)
C2—C1—C10—C9	136.8 (4)	C21—C20—C22—C23	87.2 (5)
C11—C9—C10—C19	−53.3 (5)	C17—C20—C22—C23	−140.7 (4)
C8—C9—C10—C19	77.7 (5)	C20—C22—C23—C24	39.0 (5)
C11—C9—C10—C5	−179.9 (4)	C20—O33—C24—C23	20.8 (5)
C8—C9—C10—C5	−48.9 (5)	C20—O33—C24—C25	148.5 (4)
C11—C9—C10—C1	64.0 (5)	C22—C23—C24—O33	−37.0 (5)
C8—C9—C10—C1	−165.0 (4)	C22—C23—C24—C25	−157.1 (4)
C8—C9—C11—C12	58.0 (5)	O33—C24—C25—O34	−179.1 (4)
C10—C9—C11—C12	−167.7 (4)	C23—C24—C25—O34	−59.9 (6)
C9—C11—C12—C13	−54.2 (5)	O33—C24—C25—C27	−60.4 (6)
C11—C12—C13—C17	−179.8 (4)	C23—C24—C25—C27	58.8 (6)
C11—C12—C13—C14	55.3 (5)	O33—C24—C25—C26	64.0 (6)
C12—C13—C14—C15	176.7 (4)	C23—C24—C25—C26	−176.8 (5)

### Hydrogen-bond geometry (Å, °)

**Table:**

*D*—H···*A*	*D*—H	H···*A*	*D*···*A*	*D*—H···*A*
C19—H19A···O32	0.96	2.44	3.082 (6)	124
O34—H34A···O32^i^	0.82	2.20	3.010 (5)	170
C26—H26A···O31^i^	0.96	2.53	3.392 (7)	150

Symmetry codes: (i) −*x*+1/2, −*y*+2, *z*−1/2.
